# Interprofessional collaboration and patient-reported outcomes in inpatient care: protocol for a systematic review

**DOI:** 10.1186/s13643-018-0797-3

**Published:** 2018-08-21

**Authors:** Laura Kaiser, Sina Bartz, Edmund A. M. Neugebauer, Barbara Pietsch, Dawid Pieper

**Affiliations:** 1 Witten/Herdecke University, Witten, Germany; 2 The Federal Joint Committee, Berlin, Germany; 3 Brandenburg Medical School Theodor Fontane, Neuruppin, Germany; 4 Institute for Research in Operative Medicine, Witten, Germany

**Keywords:** Interprofessional, Interdisciplinary, Collaboration, Patient-reported outcomes, Patient-reported experiences, Quality improvement, Quality of care, Inpatient

## Abstract

**Background:**

Interprofessional collaboration (IPC) is a core demand of policymakers, funding parties, and health care professionals in practice. Although the causal mechanism from increased IPC to improved patient outcomes seems to be intuitive, there is a lack of credible causal evidence concerning the effects not only on the objective but also on the subjective patient outcomes. The aim of the planned systematic review is to focus on the effect of IPC on patient-reported outcomes and experiences in inpatient care.

**Methods:**

A systematic literature review will be undertaken by searching the following electronic databases: PubMed, Web of Science/Social Science Citation Index, Cochrane Library (CENTRAL), Current Contents (LIVIVO), CINAHL, and EMBASE. Additional studies will be identified through forward and backward citation tracking, manually searching the Internet and Google Scholar, and consultation of experts. Data will be synthesized through narrative description, grouping, and thematic analysis of the extracted data. If heterogeneity for some studies and outcomes is sufficiently low, a quantitative meta-analysis of effect sizes and standard errors will be applied.

**Discussion:**

The systematic review will synthesize the evidence regarding the effectiveness of IPC and how it is perceived by patients in inpatient care. As the patients’ perspective becomes increasingly relevant in the context of quality improvement, the results can help decision-makers in policy- and health care institutions to understand and develop strategies to ensure a high quality of care.

**Systematic review registration:**

PROSPERO registration number: CRD42017073900; date of registration in PROSPERO 07 August 2017.

**Electronic supplementary material:**

The online version of this article (10.1186/s13643-018-0797-3) contains supplementary material, which is available to authorized users.

## Background

Fostering interprofessional collaboration (IPC) has become one of the core demands of policymakers, funding parties, and health care professionals in practice worldwide [1–5]. The underlying assumption of IPCs’ causal mechanism seems to be quite intuitive, suggesting that a high degree of IPC leads to better patient outcomes in terms of objective (e.g., reduced mortality rate, length of stay, or readmission) and/or subjective (patient-rated) outcomes (e.g., overall satisfaction, willingness to recommend the health care institution to others, subjective success of treatment). However, the causal mechanism remains unclear [6–10].

Additionally, credible causal evidence indicating positive patient-related outcomes is lacking and thus calling the general effectiveness of interprofessional interventions into question. One of the reasons may be that IPC can be defined as a “complex intervention,” as it consists of several possibly interacting components which make it difficult to investigate causal impacts of just one single component [11, 12]. Because of that, there is a high degree of heterogeneity in the definitions of “interprofessionalism” and so-called interprofessional interventions in the existing literature [6, 7, 12, 13] (see the “Inclusion criteria” section for the adapted definition of IPC in the planned systematic review).

**Fig. 1:**
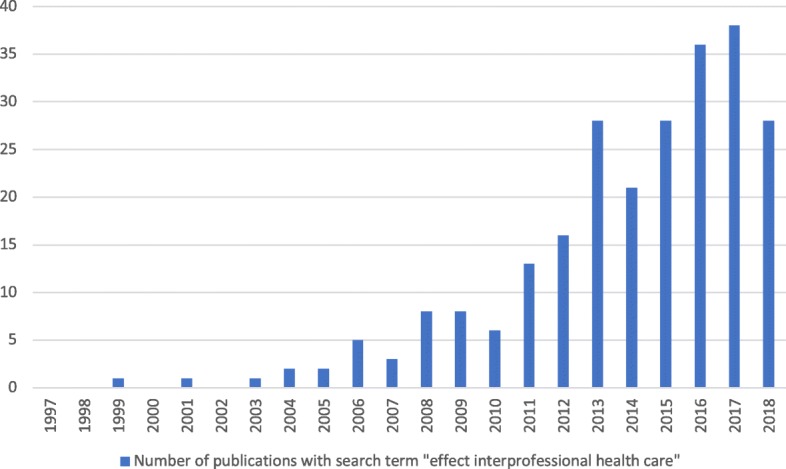
Growing body of literature (publications from 1997 till July 2018; source: SSCI, 12 July 2018)

There is a steadily growing body of literature on the effects of IPC (see Fig. 1). An early review by the Cochrane Collaboration Group [6] focuses on the effect of IPC on one single *subjective* patient-reported outcome (i.e., patient satisfaction). Unfortunately, the authors only include a small number of studies and therefore conclude that IPC “should be labeled ‘promising’ rather than ‘proven’” [6]. They recommend evaluating IPC within more rigorous evaluation studies, so that later reviews may find more substantial and credible evidence. A more recent Cochrane review from 2017 [14] examined the effect of IPC interventions on patient health outcomes, such as quality of life and patient-assessed quality of care, by reviewing randomized control trials. Once again, the authors conclude that the body of evidence is limited—only one of nine included studies referred to the *subjective* outcome of patient-assessed quality of life. Another systematic review [7] deals with the effect of IPC on *objective* patient-related outcomes. Here, the authors conclude that interprofessional interventions on general medical wards have little effects on the objective patient outcomes. However, their review does not include evidence on *subjective* and patient-reported outcomes, even though the authors state that such an analysis would be “valuable.”

Both the Cochrane review and the systematic review by Pannick et al. focus on few outcomes even though subjective outcomes are increasingly reported within the primary studies of adequate internal validity. Overall, the evidence base regarding the effects of IPC appears to be weak because of small effect sizes on objective outcomes, studies with generally only low or questionable internal validity, and omission of patient-reported outcomes in available research syntheses. Thus, the planned systematic review aims to add to the current state of literature by synthesizing evidence of IPC on (*subjective*) patient-reported outcomes and experiences in inpatient care. The results are expected to lead to relevant implications for policymakers, health care professionals, and other stakeholders by helping them to understand the patient-related effectiveness of a complex intervention like IPC.

This protocol outlines the planned systematic review in accordance with the Preferred Reporting Items for Systematic Reviews and meta-analyses Protocols (PRISMA-P) checklist (see Additional file 1) [15]. The review will focus on the following questions:

1. Does IPC affect patient-reported outcomes and patient-reported experiences in inpatient care?
2. Are there any heterogeneous effects of IPC by type of intervention?
3. Are there any heterogeneous effects of IPC within different medical fields and/or study populations?

## Methods/design

### Criteria for or against the inclusion of studies

#### Inclusion criteria

##### Types of participants

As inpatient care operates in a more controlled setting compared to ambulatory care, the review includes only the study populations of patients who received interprofessional interventions in an inpatient setting.

##### Types of interventions

As mentioned above, there is a high degree of heterogeneity in the literature regarding the definitions of IPC. In the planned systematic review, the definition of IPC is adapted from the Cochrane review [14] and the systematic review by Pannick et al. [7], as they represent the key publications in this literature. Therefore, IPC is defined as a work-sharing cooperation in which professionals from more than one health or social care profession cooperate with the explicit goal of improving collaboration and/or increasing patient-related care quality.

According to Reeves et al. [16], interprofessional interventions can be categorized into three groups: (1) interprofessional education, (2) interprofessional practice, and (3) interprofessional organization. If possible, the planned systematic review will adapt these categories to split up the described interventions in the included studies.

##### Types of included studies

The review will include randomized controlled and controlled clinical trials as well as controlled before-and-after studies and interrupted time series designs. The Cochrane Effective Practice and Organization of Care Groups’ (EPOC) criteria and terminology are used to define the different study types [17].

##### Types of outcomes

The review will include studies which report on patients’ *subjective* outcomes and experiences, such as overall satisfaction, willingness to recommend, quality of life, or subjective success of treatment. We will not distinguish between studies reporting *subjective* outcomes as primary or secondary outcomes.

#### Exclusion criteria

There are nine reasons for the exclusion of studies (see Table 1). As soon as one of the nine criteria applies to a study, it will be excluded.

**Table 1:** Exclusion criteria

Exclusion criteria	
A1	Thematically not relevant (no interprofessional collaboration as defined, no patient-reported outcomes and/or experiences)
A2	Research question not suitable (e.g., impact assessment of a complex intervention without focusing on interprofessional collaboration)
A3	Design (e.g., comment, letter to the editor)
A4	Duplicate
A5	Context not transferable (WHO mortality stratum B to E)
A6	Date of publication (date before 1997)
A7	Language (not in German or English)
A8	Full text not available
A9	Animal or laboratory study

### Search methods for identification of studies

#### Electronic databases

Relevant publications will be identified through a systematic search of the following six electronic databases:

- PubMed (see the draft in Table 2)
- Web of Science/Social Science Citation Index
- Cochrane Library (CENTRAL)
- Current Contents (LIVIVO)
- CINAHL (EBSCO)
- EMBASE

**Table 2:** PubMed search strategy (draft)

PubMed search strategy, searched on XX July 2017			
Concept	Search #	Search string	Hits
Inpatient care	1	Inpatients [MeSH]	
	2	ward* [ti,ab]	
Interprofessional collaboration (IPC)	3	interprofessional relations [MeSH]	
	4	patient care team [MeSH]	
	5	intersectoral collaboration [MeSH]	
	6	team* [ti,ab]	
	7	cooperat* [ti,ab]	
	8	co-operat* [ti,ab]	
	9	collaborat* [ti,ab]	
	10	transprofession* [ti,ab]	
	11	trans-profession* [ti,ab]	
	12	transdisciplinar* [ti,ab]	
	13	trans-disciplinar* [ti,ab]	
	14	multiprofession* [ti,ab]	
	15	multi-profession* [ti,ab]	
	16	multidisciplinar* [ti,ab]	
	17	multi-disciplinar* [ti,ab]	
	18	interprofession* [ti,ab]	
	19	inter-profession* [ti,ab]	
	20	interdisciplinar* [ti,ab]	
	21	inter-disciplinar* [ti,ab]	
Patient-reported outcomes (PRO), patient-reported experiences (PRE)	22	Patient Outcome Assessment [MeSH]	
	23	patient reported [ti,ab]	
	24	patient-reported [ti,ab]	
	25	outcom* [ti,ab]	
	26	experienc* [ti,ab]	
	27	patients’ [ti,ab]	
	28	satisfact* [ti,ab]	
	29	rating* [ti,ab]	
	30	perspect* [ti,ab]	
	31	percept* [ti,ab]	
	32	quality of life [ti,ab]	
Combination within concepts	33	1 OR 2	
	34	3 OR 4 OR 5 OR 6 OR 7 OR 8 OR 9 OR 10 OR 11 OR 12 OR 13 OR 14 OR 15 OR 16 OR 17 OR 18 OR 19 OR 20 OR 21	
	35	22 OR 23 OR 24 OR 2526 OR 27 OR 28 OR 29 OR 30 OR 31 OR 32	
Combination of concepts	36	33 AND 34 AND 35 [year 1997-2017; humans; language English or German]	

The electronic search strategy has been developed in agreement with an information professional and was checked by a researcher with long-term experience regarding systematic reviews and search strategies following the guideline for Peer Review of Electronic Search Strategies (PRESS) [18].

#### Other sources

Further studies will be found through forward and backward citation tracking as well as a manual search of the Internet and Google Scholar. Experts (as for example authors of included studies) will be consulted to identify the relevant and perhaps missing primary studies.

### Data collection

The screening process consists of two stages which are carried out by two reviewers (LK and SB): (1) title and abstract screening and (2) full-text screening. After reading a study’s title and abstract, it will be categorized into either “in” or “out” during the first screening step. If there is any concern (e.g., because of a very brief or missing abstract), the study will be categorized with “in,” to determine its eligibility in the second screening step. For each study included in the first screening, the full text will be obtained.

During the second screening (full-text screening), the group of excluded studies will be allocated to one of the nine exclusion criteria (see Table 1). All studies included in the second screening will be subject to data extraction. After finishing the full-text screening, included studies will be checked for their references, and authors will be contacted to identify possible missing studies.

To assess inter-rater reliability, a subsample of 10% of the full sample of studies will be selected and screened independently by LK and SB. If inter-rater reliability is sufficiently high (kappa statistic ≥ 0.81 [19]), subsequent screening of the remaining 90% of the full sample will be conducted by LK and SB with each coder covering 45% of the remaining sample.

### Data extraction and management

Data extraction will include country, setting (medical field), definition of IPC, description of intervention and the authors’ suggested causal mechanism, study design, study population size, participant demographics, intervention classification to one of the three intervention groups (interprofessional education, interprofessional practice, interprofessional organization) according to Reeves et al. (see above), description of intervention, details to control conditions, times of measurement, outcomes (such as overall satisfaction, willingness to recommend, quality of life, or subjective success of treatment), and robustness examinations.

**Fig. 2:**
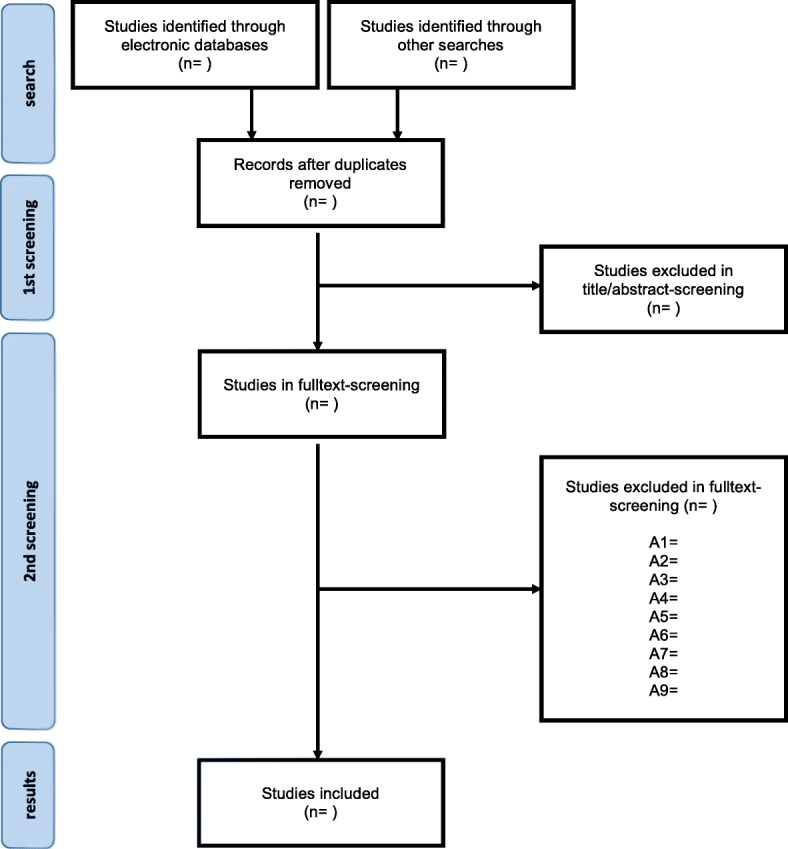
Flow diagram of systematic review

Studies excluded in the second screening will be recorded in a supplementary appendix. A flow diagram (see Fig. 2) will be used to illustrate the process of study selection and data collection.

### Quality assessment

Risk of bias of included studies will be assessed using the Cochrane “Risk of Bias” (RoB) tool for randomized controlled trials [20] and the “Risk Of Bias In Non-randomized Studies” of Interventions (ROBINS-I) [21]. If there is anything missing which is important for bias assessment, the authors will be contacted and asked to provide further information.

The EPOCs’ quality criteria will be used to assess the methodological quality of each included study. According to the EPOCs’ data collection checklist, each criterion will be rated and categorized as “done,” “not clear,” or “not done” [17].

The information of the quality assessment will be used to determine which studies are subject to a possible quantitative analysis (see below the “Data synthesis” section).

### Data synthesis

Studies will be primarily synthesized through narrative description, grouping, and thematic analysis of the extracted data.

If principle comparability for specific subsamples of studies regarding interventions, participants, and outcomes is sufficiently given, a quantitative meta-analysis of effect sizes and standard errors will be applied to the subsamples of studies with low risk of bias. Because IPC is generally a complex intervention with multiple dimensions of possible between study-heterogeneity, a random-effects meta-analysis model is the most appropriate starting point of quantitative synthesis. For the subsamples of studies on similar outcomes, interventions, and participants, a random-effects meta-analysis [22] with Knapp and Hartung corrected standard errors [23] will be conducted. Due to the expected high levels heterogeneity (with regard to study types, interventions, and study populations), pooled analysis across interventions and participant groups is not planned.

Publication bias will be examined primarily via visual inspections of funnel plots. If appropriate, an adjustment via statistical methods such as “trim and fill” [24] or the PET-PEESE procedure [25] will be evaluated as options.

The results of the systematic review will be reported and published according to the Preferred Reporting Items for Systematic Reviews and Meta-analyses (PRISMA) checklist [26].

## Discussion

The described systematic review aims to contribute to the literature on IPC and its effects on patient-reported outcomes. The effectiveness of IPC regarding the objective outcomes is still uncertain, but the available evidence clearly tilts towards small average positive effect sizes on selected objective outcomes with no available evidence regarding subjective patient-related outcomes. Thus, this systematic review tries to address this gap in the literature by synthesizing primary studies focusing on this question. It can be assumed that IPC—as a complex intervention—may have greater effects on subjective outcomes in comparison to objective outcomes.

As patients’ experiences gain more attention in terms of quality improvement in inpatient care and especially in terms of patient-perceived quality of care, it seems to be important to understand how IPC is experienced and how it effects patient-reported outcomes. Therefore, the results of this systematic review can not only widen the insights of IPC as a form of inpatient care, but also the knowledge about the patient-reported quality of care. The results are expected to lead to relevant implications for policymakers, decision-makers, and health professions in daily practice and will contribute to a balanced assessment of the effects of IPC on patient-related outcomes.

## Additional file

Additional file 1: PRISMA-P checklist. (DOCX 33 kb)

## Abbreviations

EPOC: Cochrane Effective Practice and Organization of Care Group
IPC: Interprofessional collaboration
PRESS: Peer Review of Electronic Search Strategies
